# *PLOD2* gene expression in infrapatellar fat pad is correlated with fat mass in obese patients with end-stage knee osteoarthritis

**DOI:** 10.1016/j.ocarto.2024.100469

**Published:** 2024-04-16

**Authors:** J. Van den Langenbergh, Y.M. Bastiaansen-Jenniskens, G.J.V.M. van Osch, J. Runhaar, S.M.A. Bierma-Zeinstra, K. Soballe, J. Laursen, A. Liljensoe, N. Kops, I. Mechlenburg, S. Clockaerts

**Affiliations:** a KU Leuven, Department of Development and Regeneration, Skeletal Biology and Engineering Research Center, Leuven, Belgium; b Erasmus MC, University Medical Center Rotterdam, Department of Orthopaedics and Sports Medicine, Rotterdam, Netherlands; c Erasmus MC, University Medical Center Rotterdam, Department of Otorhinolaryngology, Rotterdam, Netherlands; d Erasmus MC, University Medical Center Rotterdam, Department of General Practice, Rotterdam, Netherlands; e Aarhus University Hospital, Orthopaedic Research Unit, Aarhus, Denmark; f H.H. Z. Lier, Orthopedic Surgery and Traumatology, Lier, Belgium

**Keywords:** Fibrosis, Infrapatellar fat pad, Knee, Obesity, Osteoarthritis, *PLOD2*

## Abstract

**Objective:**

To investigate associations between obesity-linked systemic factors and gene expression indicative for the inflammatory and fibrotic processes in the infrapatellar fat pad (IFP), in a population of obese patients with end-stage knee osteoarthritis (KOA).

**Methods:**

We collected human IFPs from 48 patients with a mean body mass index (BMI) of 35.44 ​kg/m^2^ during total knee replacement procedures. These patients were part of a randomized controlled trial and met the criteria of having OA and a BMI of ≥30 ​kg/m^2^. Blood samples were collected to assess serum levels of glucose, total cholesterol, HDL cholesterol, LDL cholesterol, triglycerides, and leptin. Total body composition was measured using dual-energy X-ray absorptiometry. Gene expressions of *IL6, TNFA, COL1A1, IL1B, ASMA, PLOD2* in the IFP were analyzed.

**Results:**

Univariate analysis resulted in a positive correlation between BMI and procollagen-lysine,2-oxoglutarate 5-dioxygenase 2 (*PLOD2*) expression (*r*^2^ ​= ​0.13). In univariate analyses of obesity-linked systemic factors and *PLOD2*, significant correlations were found for lean mass (*r*^2^ ​= ​0.20), fat mass (*r*^2^ ​= ​0.20), serum cholesterol (*r*^2^ ​= ​0.17), serum triglycerides (*r*^2^ ​= ​0.19) and serum leptin (*r*^2^ ​= ​0.10). A multiple linear regression model indicated fat mass to be a strong predictor of PLOD2 production in the IFP (*r*^2^ ​= ​0.22, *P* ​= ​0.003).

**Conclusion:**

Our study demonstrates the positive association between fat mass and *PLOD2* expression in the IFP of obese end-stage knee OA patients. This may indicate that within this patient population the fibrotic process in the IFP is influenced by systemic adipose tissue, next to local inflammatory processes.

## 1 Introduction

Osteoarthritis (OA) is widely recognized as the most prevalent joint disease characterized by abnormal joint tissue remodeling driven by inflammatory mediators [1,2]. This chronic condition affects various intra-articular joint components, such as cartilage, subchondral bone, synovium, and ligaments, as well as extra-articular structures including muscles, bursae, nerves, and peri-articular fat pads. In addition to inflammation, fibrosis and synovial hyperplasia are common pathological features of OA, leading to joint stiffening and pain [[3], [4], [5], [6]]. OA results from a complex interplay between systemic and local inflammatory changes [1,2,6].

Previous studies demonstrated the relationship between obesity and OA in non-weight bearing joints with an increased risk ratio between (over)weight and the development of hand OA [[7], [8], [9], [10], [11]]. These findings therefore suggest that, beyond mechanical factors, systemic factors linked to obesity play a significant role in the pathophysiology of OA.

Obesity is known to correlate with chronic low-grade systemic inflammation [12]. The dysfunctional adipocytes and adipocyte necrosis observed in obesity lead to the infiltration of proinflammatory cells, including macrophages, mast cells, and specific T cell subtypes [13,14]. This inflammatory process is distinguished by abnormal cytokine production, elevated levels of acute-phase reactants, and the activation of a network of inflammatory signaling pathways providing a potential link to OA pathogenesis [8,[15], [16], [17]]. Epidemiological studies have identified associations between OA and obesity-linked disorders, with the most substantial relationships observed between type 2 diabetes or hyperglycemia and OA, resulting in a 43% increased overall risk [8,9,18,19]. Moreover, associations between dyslipidemia, hypercholesterolemia and OA have also been reported [19,20].

Given the intra-articular but extrasynovial location of Hoffa's fat pad or infrapatellar fat pad (IFP) within the knee joint, interest has grown for its potential role in knee OA (KOA) pathogenesis [21,22].

IFP consists of a fibrous scaffold embedded with adipose tissue, responsible for distributing synovial fluid and mechanical forces within the knee [18,21,22]. It operates as a morphofunctional unit with the synovial membrane secreting several cytokines, interleukins (ILs), adipokines and growth factors, many of which contribute to synovial fluid levels [16,[21], [22], [23], [24], [25], [26], [27], [28]]. These inflammatory mediators primarily originate from the stromal vascular fraction, consisting of fibroblasts, macrophages, leukocytes, and other cells involved in inflammatory regulation [18,24].

Interestingly, IFP possesses its own distinct inflammatory and biomechanical phenotype, setting it apart from other adipose tissues, such as subcutaneous adipose tissue and visceral adipose tissue [29,30].

Compared to ScAT, the adipose tissue within the IFP of OA patients exhibits higher IL-6, IL-8 adiponectin, adipsin, tumor necrosis factor α, visfatin levels, vascularity and increased tissue fibrosis [23,29,31].

Moreover, the OA IFP has a distinct phenotype compared to healthy subjects with thicker lobular septa, a higher grade of lymphocytic infiltration, and higher expression of IL-6 and vascular endothelial growth factor compared to patients without history of symptomatic OA [32,33].

Research exploring the impact of obesity on IFP characteristics has yielded conflicting evidence. Bastiaansen-Jenniskens et al. could not find any association between body mass index (BMI) and the presence of CD68+, CD86+ (pro-inflammatory type macrophages) and CD206+ (profibrotic type macrophages) cells in the IFP besides an increase in TNFα expression with high BMI [24,34]. Furthermore, Eymard et al. could not find any correlation among inflammatory cytokines release, fibrosis, and obesity [29].

In contrast however, Barboza et al. did find a correlation between obesity and an increase in IFP fibrosis in diet-induced obese mice and Harasymowicz et al. observed higher levels of CD45+CD14+ total macrophages and CD14+CD206+ M2 type macrophages as compared to the IFP of lean subjects [35,36].

Given these observations, we hypothesize that obesity-related systemic factors may influence the inflammatory and fibrotic responses within the IFP among obese patients with end-stage KOA. The objective of our study is to investigate these potential relationships through a secondary exploratory analysis within a cohort of obese patients scheduled for total knee replacement (TKR) based on a single-blinded, single-centre, randomized controlled trial assessing the effect of body weight reduction of 5%–10% before TKR on health-related quality of life (QOL), knee function, and body composition after surgery [37]. Our research aims to shed light on the interplay among obesity, systemic factors, and inflammation in KOA, with a particular focus on IFP.

## 2 Methods

### 2.1 Patient selection

This study is a secondary exploratory analysis based on a single-blinded, single-centre, randomized controlled trial aimed to study the effect of body weight reduction of 5%–10% before TKR on health-related QOL, knee function, and body composition after surgery [37]. Patients with OA scheduled for primary TKR, a BMI of 30 ​kg/m^2^, and motivated for weight loss were eligible for inclusion. Exclusion criteria were rheumatoid arthritis and planned bariatric surgery. Patients operated on both knees during the project period only participated once. Patients included in the diet group received a low-energy liquid diet (810 ​kcal/day) using commercially available formula foods (Cambridge Weight Plan®, Northants, UK) and nutritional education during 8 weeks pre-surgery. Patients received both oral and written information about the study in the outpatient clinic. Ethical approval was granted by Central Denmark Region Committees on Health Research Ethics (Journal number: S-201001309), and the study was registered at https://clinicaltrials.gov/(NCT01469403) [37].

### 2.2 Demographic characteristics and outcome measurements

Demographic characteristics such as age and sex were recorded before the intervention (diet group) and preoperatively (control group). For all patients, body weight was measured in kilograms on the same decimal scale (Stand weight, Kern Capacity 0–200 ​kg, class III, approved, Kern-sohn, Ballingen, Germany) wearing light clothing. Body height was measured with a digital altimeter (Soehnle 5003, Leifheit, Nassau, Germany), and BMI was calculated (kg/m^2^). As primary outcome the patient-reported outcome measure Short-Form 36 (SF-36) subscale Physical function Component Score was chosen. Secondary outcomes were SF-36 subscales Mental Component Score, Knee injury and Osteoarthritis Outcome Score (KOOS), and 6-Min Walk Test (6 ​MW). All PROMs were reported electronically by patients into the project database. The 6 ​MW was based on standard procedures on a 30-m course marked for every 5 ​m under supervision of a project physiotherapist.

### 2.3 Body composition

In addition, total body composition (fat mass, lean mass) was measured using dual-energy X-ray absorptiometry (DXA). DXA (Lunar Prodigy Advance®, GE Healthcare, Chicago, IL, USA) narrow-angle fan beam is a non-invasive, precise, and operator independent method that exposes the patient to a low radiation dose. All scans were analyzed using the enCORE® software, version 13.60 (GE Healthcare, Chicago, IL, USA). The scanner's calibration was checked daily before each scanning session, using the GE Lunar calibration phantom and the manufacturer's guidelines for patient positioning and for scan acquisition were strictly followed. To measure the variance of the total body scans, double scanning was performed on all the project participants and the coefficient of variation was 0.1%.

### 2.4 Serum samples

Blood samples (non-fasting) were collected to assess glucose, lipid status (total cholesterol (TC), high-density lipoprotein cholesterol (HDL), low-density lipoprotein cholesterol (LDL), and triglyceride (TGLY). In addition, serum leptin concentration was measured as a biomarker of adiposity (28). Non-fasting samples were chosen to simplify blood sampling. Recent literature provided evidence that levels of lipids, lipoproteins, and apolipoproteins after normal food intake only differ minimally from levels in the fasting state [38].

### 2.5 RNA isolation and quantitative RT-PCR

IFPs of study participants were obtained as leftover material during the TKR. The fat pads were frozen at −80 ​°C within 1 ​h after being removed. Frozen IFP samples were homogenized with a Mikro-Dismembrator (Braun Biotech International GmbH, Melsungen, Germany) and suspended in 1.8 ​mL per 100 ​mg tissue RNA-Bee (Bioconnect). The RNA-bee solution was precipitated with 400 ​μL chloroform. RNA was purified using a Rneasy Micro Kit (Qiagen, Hilden, Germany). A 1000 ​ng of total RNA was reverse-transcribed into cDNA using RevertAid First Strand cDNA synthesis Kit (MBI Fermentas, St. Leon-rot, Germany). By means of PrimerExpress 2.0 software (Applied Biosystems, Foster City, CA, USA), forward and reverse primers for the real-time (RT-PCR) reaction were designed to meet Taqman (ABI, Branchburg, New Jersey) requirements, and to bind the separate exons to avoid amplification of genomic DNA. The designed primer sequences were run against a genome-wide database (BLASTN) to ensure gene specificity of the primers and probes.

The following primers were used: procollagen-lysine,2-oxoglutarate 5-dioxygenase 2 (*PLOD2*) involved in the regulation of hydroxyallysine collagen crosslinks, alpha-smooth muscle actin (*ASMA*) corresponding with the myofibroblast phenotype, collagen type 1, alpha 1 (*COL1A1*) as markers of fibrosis, and interleukin 6 (*IL6*), tumor necrosis factor alpha (*TNFA*), interleukin 1 ​bèta (*IL1B*) as markers of inflammation. *GAPDH*, Probe: CGCCCAATACGACCAAATCCGTTGAC GenBank accession no. NM_002046.3) was used as housekeeping gene to calculate relative gene expression by means of the 2deltaCT formula [39]. Ct GADPH and BMI, pre-op fatmass, and pre-op weight did not correlate (*r*^2^ ​< ​0.09. Supplementary Table S2 lists the corresponding forward and reverse oligonucleotides used. TaqMan Universal PCR Master Mix (ABI, Branchburg, New Jersey) or qPCR™ Mastermix Plus for SYBR®Green I (Eurogentec, Nederland B.V., Maastricht, The Netherlands) was used to perform (RT-PCR) in 10 ​μl reactions according to the manufacturer's guidelines and using an ABI PRISM 7000 with SDS software, version 1.2.3.

### 2.6 Statistical analysis

Power analysis for the clinical trial was performed to obtain 80% power to detect an 8% difference between groups in the SF-36 physical component score 12 months after TKR in the clinical trial [37]. We did not perform an additional power analysis to detect significant results in IFP characteristics.

A Shapiro-Wilk test was performed to test for normal data distribution.

Both diet and control group patients were mixed for our study purpose after paired *t*-tests ruled out significant differences in IFP characteristics (*PLOD 2, ASMA, COL1A1, IL6, TNFA, IL1B*) between diet and control group (Table 2). To determine correlations between obesity-linked systemic factors and IFP characteristics based on gene expression a Pearson's correlation test was performed. Correlations were withheld as significant with a P value ​≤ ​0.05. Correlation plot on patient characteristics was performed to check for collinearity, where a correlation coefficient of 0.5 was considered the threshold for exclusion. Based on this, a multiple linear regression analysis was performed to further analyze the correlations between these selected independent variables and *PLOD2*. All analyses were performed in Graphpad Prism 9® (San Diego, CA, USA).

**Table 1:** Unpaired *t*-test comparing pre-operative patient demographics in control and diet group.

Pre-operative patient demographics control vs. diet group	Mean control group	Mean diet group	*P* value	95% CI
Age	65.58 ​y	65.37 ​y	*P* ​= ​0.94	CI ​= ​−5.69 to 5.29
BMI	37.52 ​kg/m^2^	33.68 ​kg/m^2^	*P* ​= ​0.009	CI ​= ​1.02 to 6.67
Weight	106.1 ​kg	93.5 ​kg	*P* ​= ​0.002	CI ​= ​4.76 to 20.41
Fat mass	49.58 ​kg	40.48 ​kg	*P* ​= ​0.003	CI ​= ​3374 to 14817
Serum glucose	6.26 ​mmol/l	5.94 ​mmol/l	*P* ​= ​0.47	CI ​= ​−0.581 to 1.23
Serum total cholesterol	5.06 ​mmol/l	4.07 ​mmol/l	*P* ​= ​0.004	CI ​= ​0.34 to 1.64
Serum HDL cholesterol	1.44 ​mmol/l	1.34 ​mmol/l	*P* ​= ​0.37	CI ​= ​−0.12 to 0.31
Serum LDL cholesterol	2.85 ​mmol/l	2.26 ​mmol/l	*P* ​= ​0.06	CI ​= ​−0.03 to 1.20
Serum triglycerides	1.76 ​mmol/l	1.09 ​mmol/l	*P* ​= ​0.001	CI ​= ​0.28 to 1.06
Serum leptin	6956 ​mmol/l	33.6 ​mmol/l	*P* ​< ​0.001	CI ​= ​17.05 to 54.88

CI ​= ​95% confidence interval, P ​= ​P value of statistical significance.

**Table 2:** Unpaired *t*-test comparing IFP characteristics of diet group and control group.

Gene expression	*P* value	t,df	Mean of control group	Mean of dietgroup	95% CI	R squared	SD control group	SD diet group
PLOD2	0.17	t ​= ​1.40, df ​= ​46	0.005	0.004	−0.003 to <0.001	0.04	0.003	0.002
COL1A1	0.60	t ​= ​0.52, df ​= ​46	7.21	8.27	−3.01 to 5.14	0.006∗	6.08	7.67
ASMA	0.41	t ​= ​0.83, df ​= ​46	3.68	3.13	−1.89 to 0.79	0.01	2.84	1.70
IL1B	0.09	t ​= ​1.73, df ​= ​46	0.006	0.004	−0.005 to <0.001	0.06	0.006	0.002
TFNA	0.34	t ​= ​0.97, df ​= ​46	0.03	0.03	−0.04 to 0.01	0.02∗	0.008	0.01
IL6	0.20	t ​= ​1.29, df ​= ​46	0.02	0.01	−0.03 to 0.007	0.03∗	0.04	0.02

Gene expression normalized for GADPH. ∗ ​= ​negative correlations.

## 3 Results

### 3.1 Patients

A total of 48 IFP specimens of 48 patients were collected between October 2011 and May 2013. Diet group consisted of 22 patients and the control group consisted of 26 patients. Patients mean age was 64.91 years (SD 9.23) with a mean BMI of 35.44 ​kg/m^2^ (SD 5.11) (mean height of 167.6 (SD 9.94) cm and a mean weight of 99.27 ​kg (SD 14.56). Six patients, all part of the diet group, had a BMI >25 ​kg/m^2^ and <30 ​kg/m^2^. Mean fat mass was 44.65 ​kg. There was no equal distribution for sex (12 men, 36 women). Except for age (*P* ​= ​0.001) and LDL/HDL ratio (*P* ​= ​0.04) our data had a normal distribution. Comparison between patient demographic data is summarized in Table 1. Significant differences were observed between BMI (*P* ​= ​0.009), weight (*P* ​= ​0.002), fat mass (*P* ​= ​0.003), serum TC (*P* ​= ​0.004), Serum LDL (*P* ​= ​0.06), serum TGLY (*P* ​= ​0.001) and serum leptin (*P* ​< ​0.001).

### 3.2 *PLOD2* levels correlate with fat mass in IFP

**Fig. 1:**
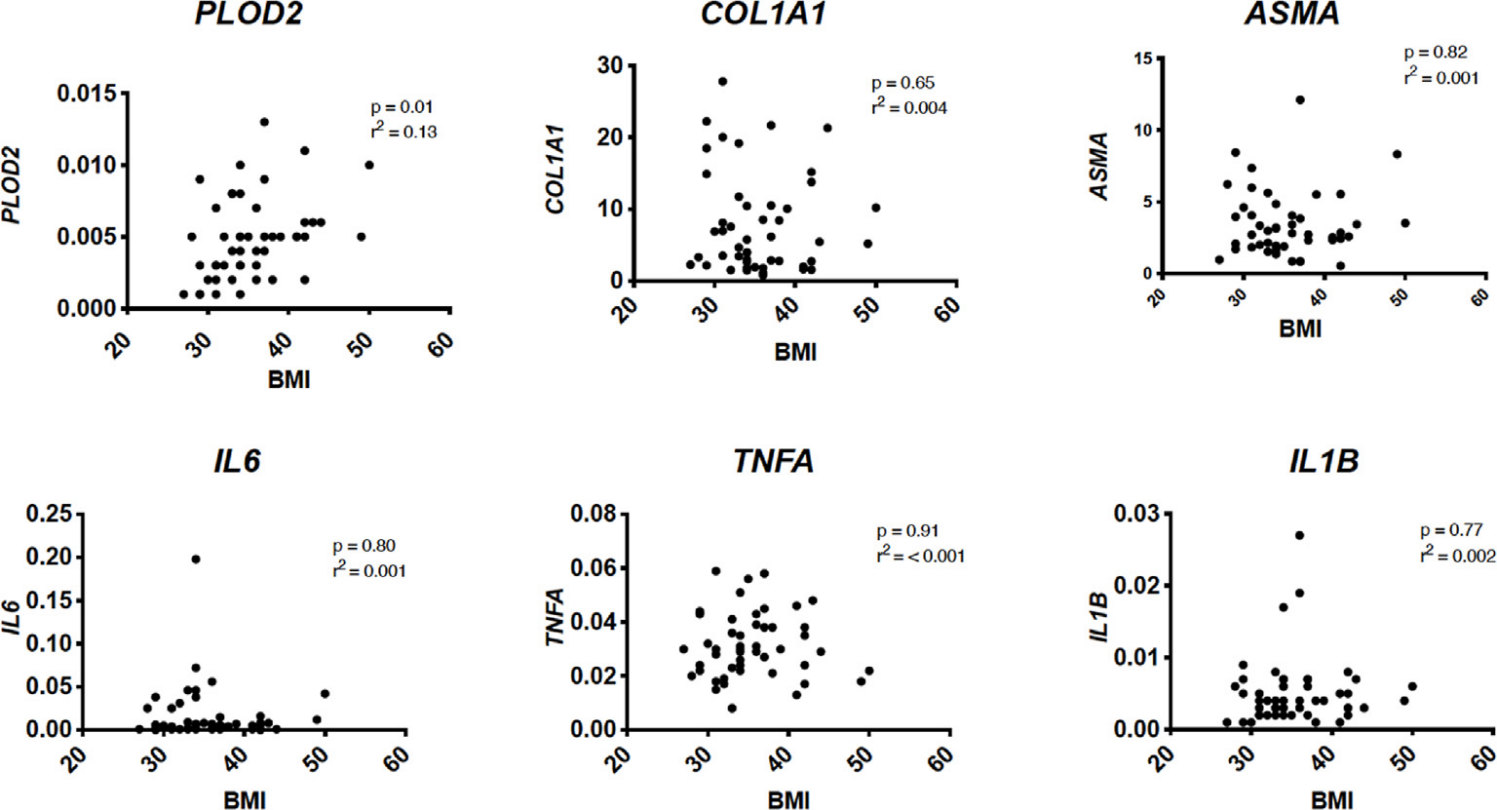
Gene expression scatter plots of determinants of fibrosis PLOD2, COL1A1, ASMA and inflammation IL6, TNFA and IL1B in correlation with BMI with CT values normalized to GADPH. P=P value of statistical significance, *r*^2^ ​= ​R squared.

Analyses of univariate correlations between BMI and IFP characteristics based on gene expression, resulted in a significant (*P* ​= ​0.01) correlation between BMI and *PLOD2* expression with 13% of the variance explained by this variable (*r*^2^ ​= ​0.13). (Fig. 1).

**Fig. 2:**
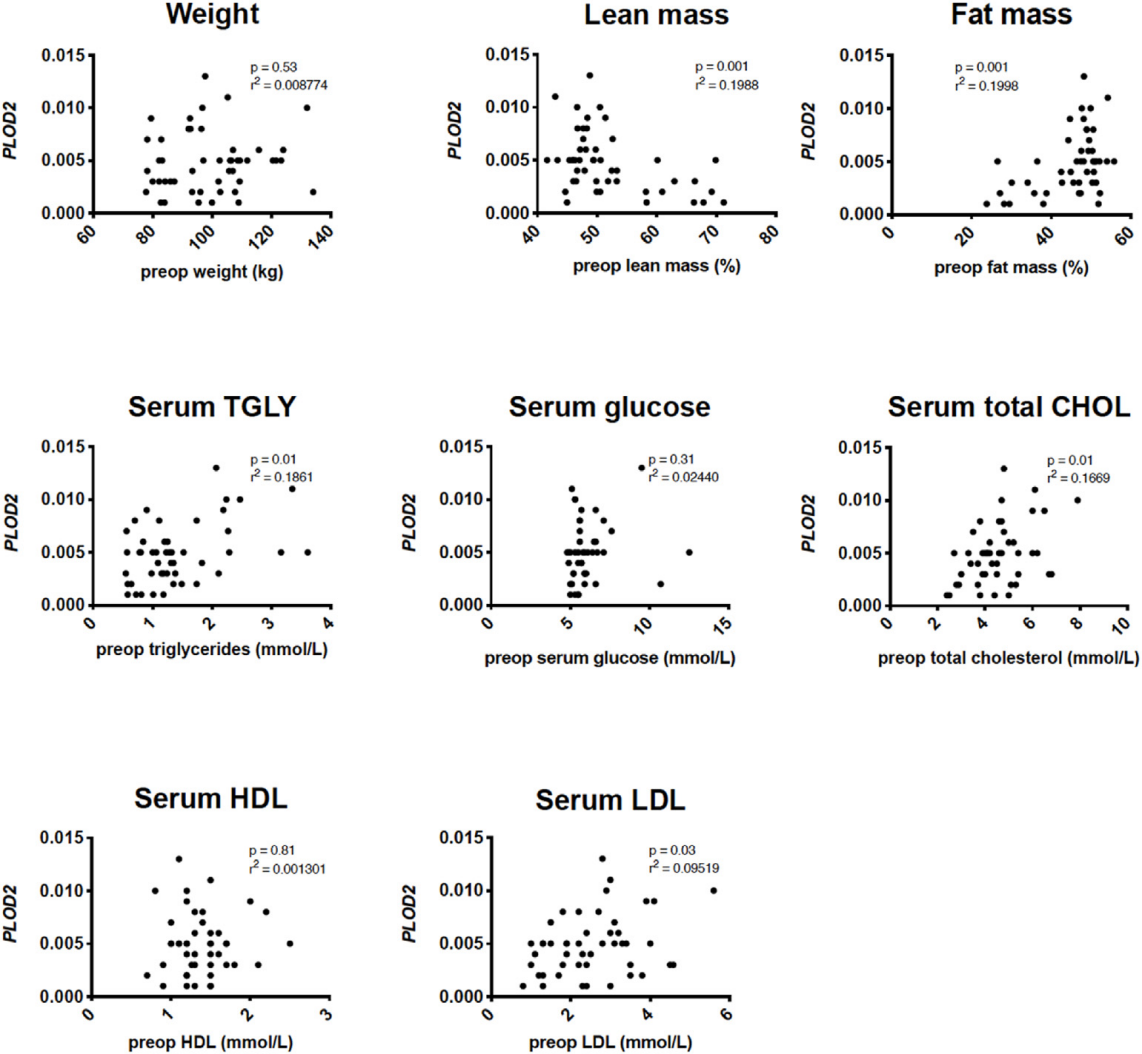
PLOD2 gene expression (CT values normalized to GADPH) in correlation with obesity-linked systemic factors in IFP of obese end-stage KOA patients presented in scatter plots. P ​= ​P value of statistical significance, *r*^2^ ​= ​R squared.

To further explore the correlation between BMI and *PLOD2*, metabolic factors associated with obesity were analyzed for their correlation with *PLOD2*. In the univariate analyses, significant correlations with *PLOD2* expression were found for lean mass, (*r*^2^ ​= ​0.20, *P* ​= ​0.002), fat mass (*r*^2^ ​= ​0.20, *P* ​= ​0.001), serum TC (*r*^2^ ​= ​0.17, *P* ​= ​0.004), serum TGLY (*r*^2^ ​= ​0.19, *P* ​= ​0.003), and serum leptin (*r*^2^ ​= ​0.10, *P* ​= ​0.03). (Fig. 2).

A collinearity plot was performed to investigate the correlation between obesity-linked systemic factors before performing a multiple linear regression analysis. We withhold age, sex, glucose, fat mass, TC as independent variables (threshold for collinearity: 0.5). (Supplementary Table 1).

The multiple linear regression analysis indicated that only fat mass remained independently associated with *PLOD2* (*r*^2^ ​= ​0.22, *P* ​= ​0.0025). No other significant correlations were found (Table 4).

**Table 3:** Pearson correlation test between IPF characteristics and obesity-linked systemic factors.

	BMI	Lean mass	Fat mass	Glucose	Total cholesterol	LDL/HDL ratio	TGLY	Leptin
Gene expression								
*PLOD2*	*r*^2^ ​= ​0.13 *P* ​= ​0.01 CI ​= ​0.09 to 0.59	*r*^2^ ​= ​0.20∗ *P* ​= ​0.002 CI ​= ​−0.65 to −0.19	*r*^2^ ​= ​0.20 *P* ​= ​0.01 CI ​= ​0.17 to 0.65	*r*^2^ ​= ​0.02 *P* ​= ​0.30 CI ​= ​−0.14 to 0.43	*r*^2^ ​= ​0.17 *P* ​= ​0.004 CI ​= ​0.14 to 0.62	*r*^2^ ​= ​0.05 *P* ​= ​0.11 CI ​= ​−0.06 to 0.49	*r*^2^ ​= ​0.19 *P* ​= ​0.003 CI ​= ​0.16 to 0.64	*r*^2^ ​= ​0.10 *P* ​= ​0.03 CI ​= ​0.04 to 0.56
*COL1A1*	*r*^2^ ​= ​0.004∗ *P* ​= ​0.65 CI ​= ​−0.34 to 0.22	*r*^2^ ​= ​0.04 *P* ​= ​0.15 CI ​= ​−0.08 to 0.47	*r*^2^ ​= ​−0.20∗ *P* ​= ​0.16 CI ​= ​−0.46 to 0.08	*r*^2^ ​< ​0.001 *P* ​= ​0.93 CI ​= ​−0.28 to 0.31	*r*^2^ ​= ​0.005∗ *P* ​= ​0.65 CI ​= ​−0.35 to 0.22	*r*^2^ ​= ​0.004∗ *P* ​= ​0.66 CI ​= ​−0.35 to 0.23	*r*^2^ ​= ​0.05∗ *P* ​= ​0.13 CI ​= ​−0.48 to 0.07	*r*^2^ ​= ​0.05∗ *P* ​= ​0.15 CI ​= ​−0.47 to 0.08
*ASMA*	*r*^2^ ​= ​0.001 *P* ​= ​0.82 CI ​= ​−0.25 to 0.32	*r*^2^ ​< ​0.001 *P* ​= ​0.86 CI ​= ​−0.26 to 0.31	*r*^2^ ​< ​0.001∗ *P* ​= ​0.86 CI ​= ​−0.31 to 0.26	*r*^2^ ​= ​0.006 *P* ​= ​0.61 CI ​= ​−0.22 to 0.36	*r*^2^ ​= ​0.06 *P* ​= ​0.09 CI ​= ​−0.04 to 0.50	*r*^2^ ​= ​0.008 *P* ​= ​0.55 CI ​= ​−0.20 to 0.37	*r*^2^ ​= ​0.006 *P* ​= ​0.60 CI ​= ​−0.21 to 0.36	*r*^2^ ​= ​0.01 *P* ​= ​0.47 CI ​= ​−0.18 to 0.38
*IL6*	*r*^2^ ​= ​0.001 *P* ​= ​0.80 CI ​= ​−0.32 to 0.25	*r*^2^ ​= ​0.02 *P* ​= ​0.30 CI ​= ​−0.14 to 0.42	*r*^2^ ​= ​0.02∗ *P* ​= ​0.31 CI ​= ​−0.41 to 0.14	*r*^2^ ​= ​0.01∗ *P* ​= ​0.44 CI ​= ​−0.40 to 0.18	*r*^2^ ​= ​0.001 *P* ​= ​0.83 CI ​= ​−0.26 to 0.32	*r*^2^ ​< ​0.001 *P* ​= ​0.98 CI ​= ​−0.28 to 0.29	*r*^2^ ​= ​0.007∗ *P* ​= ​0.58 CI ​= ​−0.36 to 0.21	*r*^2^ ​= ​0.02∗ *P* ​= ​0.39 CI ​= ​−0.40 to 0.16
*TFNA*	*r*^2^ < 0.001∗ *P* ​= ​0.92 CI ​= ​−0.30 to 0.27	*r*^2^ ​= ​0.002 *P* ​= ​0.77 CI ​= ​−0.24 to 0.32	*r*^2^ ​= ​0.001∗ *P* ​= ​0.81 CI ​= ​−0.32 to 0.25	*r*^2^ ​< ​0.001∗ *P* ​= ​0.93 CI ​= ​−0.31 to 0.28	*r*^2^ ​= ​0.13∗ *P* ​= ​0.01 CI ​= ​−0.59 to −0.08	*r*^2^ ​= ​0.14∗ *P* ​= ​0.01 CI ​= ​−0.59 to −0.09	*r*^2^ ​= ​0.03∗ *P* ​= ​0.22 CI ​= ​−0.45 to 0.11	*r*^2^ ​= ​0.02 *P* ​= ​0.38 CI ​= ​−0.16 to 0.40
*IL1B*	*r*^2^ ​= ​0.002∗ *P* ​= ​0.78 CI ​= ​−0.25 to 0.32	*r*^2^ ​= ​0.009 *P* ​= ​0.52 CI ​= ​−0.19 to 0.37	*r*^2^ ​= ​0.007∗ *P* ​= ​0.56 CI ​= ​−0.36 to 0.20	*r*^2^ ​= ​0.03∗ *P* ​= ​0.28 CI ​= ​−0.44 to 0.13	*r*^2^ ​= ​0.02∗ *P* ​= ​0.42 CI ​= ​−0.39 to 0.17	*r*^2^ ​< ​0.001 *P* ​= ​0.92 CI ​= ​−0.27 to 0.30	*r*^2^ ​= ​0.008∗ *P* ​= ​0.54 CI ​= ​−0.37 to 0.20	*r*^2^ ​< ​0.001∗ *P* ​= ​0.85, CI ​= ​−0.31 to 0.26

CI ​= ​95% confidence interval, P ​= ​P value of statistical significance, *r*^2^ ​= ​R squared, ∗ ​= ​negative correlations.

**Table 4:** Multiple linear regression analysis of the correlation between the independent variables and PLOD gene expression in IFP.

Variable	Estimate	Standard error	95% confidence interval	*P* Value	P value summary
Intercept	−0.002	0.0048	−0.012 to 0.0079	0.71	ns
A: age	0.038	0.044	−0.00006 to 0.00012	0.52	ns
B: sex	−0.001	0.0010	−0.0028 to 0.0013	0.45	ns
C: Fat mass	0.0002	0.051	0.00006 to 0.00026	0.0025	**∗**
D: glucose	−0.000078	0.00027	−0.00062 to 0.00047	0.78	ns
E: Total cholesterol	−0.0002	0.00035	−0.0009 to 0.0005	0.56	ns

*P* ​= ​value of statistical significance, ns ​= ​not significant, ∗ ​= ​significant.

### 3.3 *TNFA* levels correlate with TC and LDL/HDL levels in IFP

Analyses of univariate correlations between IPF characteristics and metabolic factors associated with obesity resulted in a significant correlation between *TNFA* and TC (*r*^2^ ​= ​0.13, *P* ​= ​0.01), LDL (*r*^2^ ​= ​0.13, *P* ​= ​0.01) and LDL/HDL ratio (*r*^2^ ​= ​0.14, *P* ​= ​0.01). No significant correlation was withheld between *TNFA* and HDL (*r*^2^ ​< ​0.001, *P* ​= ​0.84) (Table 3). Further multiple linear regression analysis could not withhold TC as an independent variable.

### 3.4 Correlations between IFP characteristics and PROMs

Univariate analysis between IPF characteristics and KOOS, subscores for pain, symptom, and QOL and SF-36 PCS subscale showed a significant correlation between *ILB1* and pre-operative Knee injury and Osteoarthritis Outcome Score (KOOS) sub score for symptoms (*r* ​= ​0.33, *P* ​= ​0.02). Noteworthy are also the near significant negative correlations between *PLOD2* and pre-operative KOOS, subscore for pain (*r* ​= ​−0.24, *P* ​= ​0.09) and SF36-PCS (*r* ​= ​−0.18, *P* ​= ​0.09) (Table 5).

**Table 5:** Gene expression of IFP characteristics (PLOD2, COL1A1, ASMA, IL6, TNFA, IL1B) in correlation with patient reported outcome measures.

Gene expression	Pre-op KOOS-QOL	Pre-op KOOS-symptom	Pre-op KOOS-pain	Pre-op SF36-PCS
*PLOD2*	*r* ​= ​−0.03, *P* ​= ​0.81 CI ​= ​−0.31 to 0.25	*r* ​= ​−0.03, *P* ​= ​0.86 CI ​= ​−0.31 to 0.26	*r* ​= ​−0.24, *P* ​= ​0.09 CI ​= ​−0.49 to 0.04	*r* ​= ​−0.18, *P* ​= ​0.09 CI ​= ​−0.44 to 0.11
*COL1A1*	*r* ​= ​0.07, *P* ​= ​0.60 CI ​= ​−0.21 to 0.35	*r* ​= ​0.05, *P* ​= ​0.71 CI ​= ​−0.23 to 0.33	*r* ​= ​0.09, *P* ​= ​0.53 CI ​= ​−0.20 to 0.37	*r* ​= ​0.03, *P* ​= ​0.80 CI ​= ​−0.25 to 0.32
*ASMA*	*r* ​= ​0.17, *P* ​= ​0.24 CI -0.12 to 0.44	*r* ​= ​0.01, *P* ​= ​0.93 CI ​= ​−0.27 to 0.29	*r* ​= ​−0.02, *P* ​= ​0.90 CI ​= ​−0.30 to 0.27	*r* ​= ​−0.10, *P* ​= ​0.40 CI ​= ​−0.38 to 0.18
*IL6*	*r* ​= ​−0.14, *P* ​= ​0.36 CI ​= ​−0.40 to 0.15	*r* ​= ​0.05, *P* ​= ​0.73 CI ​= ​−0.24 to 0.33	*r* ​= ​−0.05, *P* ​= ​0.71 CI ​= ​−0.33 to 0.23	*r* ​= ​−0.09, *P* ​= ​0.52 CI ​= ​−0.37 to 0.20
*TFNA*	*r* ​= ​0.07, *P* ​= ​0.65 CI ​= ​−0.22 to 0.35	*r* ​= ​0.14, *P* ​= ​0.34 CI ​= ​−0.15 to 0.41	*r* ​= ​0.13, *P* ​= ​0.39 CI ​= ​−0.16 to 0.40	*r* ​= ​0.05, *P* ​= ​0.74 CI ​= ​−0.24 to 0.33
*IL1B*	*r* ​= ​−0.002, *P* ​= ​0.99 CI ​= ​−0.29 to 0.28	*r* ​= ​0.33, *P* ​= ​0.02 CI ​= ​0.05 to 0.56	*r* ​= ​0.03, *P* ​= ​0.83 CI ​= ​−0.25 to 0.31	*r* ​= ​−0.03, *P* ​= ​0.84 CI ​= ​−0.31 to 0.26

Pre-operative knee osteoarthritis outcome score ​= ​pre-op KOOS, sub scores for pain, symptom, and quality of life (QOL), Short-Form 36 (SF-36) subscale Physical function Component Score (PCS).

## 4 Discussion

IFP has been established as a significant contributor to the pathogenesis of KOA, acting as a morphofunctional unit in conjunction with the synovial membrane [22]. It has the capacity to influence the inflammatory and fibrotic processes associated with KOA by emitting a range of cytokines, growth factors, and adipokines.

Obesity is known to correlate with a chronic low-grade systemic inflammation providing a potential link to OA pathogenesis [8,[15], [16], [17]]. Epidemiological studies have identified associations between OA and obesity-linked disorders [8,9,18,19]. At present, research findings regarding the association among inflammatory, fibrotic responses of IFP and obesity in KOA are contradictory. This study investigated associations between obesity-linked systemic factors and gene expression indicative for the inflammatory and fibrotic processes in the IFP, in a population of obese patients with end-stage KOA. The main finding of our analyses is the significant correlation between *PLOD2* in IFP of obese end-stage KOA patients and BMI, mainly explained by the percentage of total body fat mass. To our knowledge, this study is the first to demonstrate the positive association between fat mass and *PLOD2* in the IFP.

*PLOD2* codes for lysyl hydroxylase 2b (LH2b), an enzyme responsible for the hydroxylation of the lysine end of the collagen telopeptide to a hydroxylysine end increasing the amount of pyridinoline cross-links in collagen fibers [40]. LH2b is a hallmark for fibrosis and the only lysyl hydroxylase consistently up-regulated in several forms of fibrosis [41]. The key inducer of PLOD2 is transforming growth factor bèta (TGF-β). Similarly, hypoxia induced factor 1α (HIF1α), interleukin-4 (IL4), bone morphogenetic protein 2, activin A, prostaglandin F-2α (PGF2α) and tumor necrosis factor α can induce PLOD2 expression [25,42].

Recent findings demonstrated the importance of *PLOD2* in the whole pathogenesis and regulation of the fibrotic process [41,43]. Remst et al. already demonstrated the up-regulation of *PLOD2* in osteoarthritic OA human synovial fibroblasts [44].

These findings are consistent with the positive association found between obesity and an increased expression of genes (*SPARC, COL4, COL6)* associated with fibrosis and ECM production in a diet-induced obesity mice model [35]. Harasymowicz et al. also withhold positive correlations between obesity and pro-fibrotic factors in the IFP with a rise in CD14+CD206+ cells [36]. The latter are known to act as an important source of the profibrotic factor TGF-β. Furthermore, they did not find correlations in the extent of pericellular fibrosis between lean and obese patients which is opposed to the results of Favero et al. who did find a correlation between BMI and the thickness of interlobular septae [33,36]. Our findings indicate that fat mass can alter the fibrotic process in IFP by excessive hyperhydroxylation of collagen fiber making them less vulnerable to degradation.

The primary route for triggering *PLOD2* up-regulation appears to be influenced by its most potent inducer, TGF-β [45]. However, it is noteworthy that *in vitro* investigations by Bastiaansen-Jenniskens, involving fat-conditioned medium from OA IFP samples, did not yield significant alterations in *PLOD2* expression upon TGF-β inhibition, but showed PGF_2α_ to be capable of inducing *PLOD2* gene expression [25]. Additionally, Belluzzi et al. revealed lower levels of *TGF-β* gene expression in the IFP of end-stage OA patients compared to patients undergoing anterior cruciate ligament repair supporting the idea that *PLOD2* expression in the OA IFP is altered by other pathways than *via* TGF-β [32].

Despite the positive associations found with *PLOD2*, no correlations between obesity related systemic factors and other determinants of fibrosis, *COL1A1* or *ASMA* were detected. *COL1A1* encodes for α1 chain of type 1 collagen, which is the main collagen found in fibrotic disease. *COL1A1* is up-regulated in synovial tissue but seems to be down-regulated in the IFP from patients with end-stage KOA [32]. *ASMA* mRNA levels correspond with the myofibroblasts phenotype and are up-regulated in fibrotic cell populations [46]. They regulate connective tissue remodeling by combining the ECM–synthesizing features of fibroblasts with cytoskeletal characteristics of contractile smooth muscle cells [46]. The positive correlation between fat mass and PLOD2 gene expression and absence of correlation between COL1A1 gene expression hypothesizes that fat mass alters the fibrotic process in IFP by excessive hyperhydroxylation rather than a raise in collagen production nor connective tissue remodeling.

Previous research found positive correlations between the pro-inflammatory cytokine *TNFA* gene expression and BMI in human KOA IFP's and in mice OA model fed with a high fat diet [24,30,47]. Our study failed to reproduce this correlation between BMI and *TNFA* gene expression. The study of de Jong et al. with human IFP samples included 106 patients with a mean BMI of 29 ​kg/m^2^ whereas Klein-Wieringa had a much smaller sample size of 27 patients with a mean BMI of 31 ​kg/m^2^ [27,30]. BMI and sample size from our study is comparable to previous similar studies. To date we have no clear explanation why we could not reproduce those correlations. Also, only significant correlations between *TNFA* and serum levels of TC, LDL and LDL/HDL ratio were found. No previous research has been done looking for associations between *TNFA* gene expression in the IFP and serum TC, LDL levels. Indirect support for the correlation of TC in homeostasis and homeostasis in KOA is evident through the protective impact of statin usage on the progression of KOA [48].

No associations between the expression of the pro-inflammatory cytokine genes *IL6, IL1B*, and obesity-linked systemic factors were withheld. *IL6* is known to be up-regulated in the IFP of obese end-stage KOA patients in relation to both IFP of patients scheduled for ligamentous repair and SCAT [23,24,32,49]. In contrast to our results Klein-Wieringa et al. and Belluzzi et al. demonstrated a positive correlation between *IL6* gene expression and BMI [24,32]. Whereas Asou et al. did find raised *IL6* levels in the IFP of a high fat diet induced OA mice model [50].

In contrast to *IL6, IL1B* is not up-regulated in the IFP of end-stage KOA patients which can explain why our study could not find any correlation between obesity-linked systemic factors and *IL1B* gene expression [23,49].

To summarize, our study failed to reproduce a positive correlation between BMI and pro-inflammatory cytokine gene expression in the IFP of obese end-stage KOA patients was demonstrated in previous studies.

Analysis of PROMs and IFP characteristics found positive correlations between *IL1B* gene expression and the pre-operative KOOS subscore for symptoms. IL-1β is a highly potent inducer of cartilage degradation and known to be a mechanical hyperalgesic agent upon injection in peripheral tissue [51,52]. The exact pathways by whom *IL1B* acts on the symptoms in end-stage KOA have to be further elucidated. To our knowledge we are the first to present correlation data on IFP characteristics and PROMs. Studies correlating PROMs and cytokine gene expression in KOA patients are only available on synovial fluid samples. Larsson et al. could find a correlation with TNFα and KOOS subscale activity of daily living in a group of KOA patients after meniscectomy whereas Orita et al. demonstrated positive correlations between TFNα and WOMAC scores in KOA patients [53,54]. Extensive research on the relationship between pain and IFP inflammation and fibrosis has been done by the group of H. Koga et al. in a monoiodoacetic acid-induced rat knee arthritis model. They demonstrated that fibrotic changes in the IFP after joint inflammation are closely correlated with persistent joint pain [55]. Furthermore, they could alleviate joint pain by intra-articular injection of C-type natriuretic peptide before and after the occurrence of fibrosis [56]. These findings highlight the important interplay between IFP fibrosis and pain, function in KOA.

The strength of our study resides in its analysis of the interactions between IFP characteristics in obese end-stage KOA patients and the individual systemic factors commonly associated with obesity. Previous studies have predominantly focused on establishing correlations between IFP traits and BMI. However, it is essential to recognize that BMI provides a less adequate insight into body composition than DXA.

We recognize that our study has some limitations. First, we have no control group of non-obese end-stage KOA patients to compare the relationship of pro-inflammatory and pro-fibrotic gene expression in the IFP and obesity-linked systemic factors. Second, it is worth noting that gene expression can be influenced by dietary restrictions. To investigate diet-induced shifts in gene expression, collecting IFP samples before start of a low-energy diet would have been required.

Third, we did not measure gene expression in serum samples nor in visceral adipose tissue or subcutaneous adipose tissue. Therefore, we can't exclude a more overall effect of obesity and obesity related systemic factors on levels of *PLOD2*, *ASMA, COL1A1, IL6, IL1B, TNFA.* Fourth, our study is limited to quantitative gene expression analyses. Therefore, no further conclusions can be made on protein level since gene expression does not always reflect levels of protein. Finally, we did not perform a pre-study power analysis. Therefore, it is possible that we were underpowered to detect other potentially significant correlations.

## 5 Conclusion

Our study is the first to demonstrate the positive association between fat mass and *PLOD2* gene expression, a hallmark for fibrosis, in the IFP in a population of 48 obese end-stage KOA patients. These findings support the existing evidence that obesity contributes to a pro-fibrotic environment within the IFP of individuals with end-stage KOA.

Additional research is needed to unveil the systemic influences on, and the distinct contributions of, various stromal vascular fraction cell types to cytokine production within pad IFP. Likewise, further investigation is required to understand the pathways governing PLOD2 regulation in the IFP and the role of body fat mass in this process. This exploration should encompass both obese and non-obese populations of patients with end-stage OA.

## Role of funding resources

This study was funded by The Danish Rheumatism Association; Cambridge Weight Plan®, Northants, UK; Linak A/S; Fabrikant Mads Clausen's foundation Danfoss; Johs. M. Klein og hustru's foundation; Knud and Edith Eriksen's foundation; Peter Ryholt's foundation; and Jeppe Juhl og hustru Ovita Juhl's foundation. None of the sponsors were involved in the design of the study; in the collection, analysis, and interpretation of data; in writing of the manuscript; or in the decision to submit the manuscript for publication.

## Ethical approval

Ethical approval was granted by Central Denmark Region Committees on Health Research Ethics (Journal number: S-201001309), and the study was registered at https://clinicaltrials.gov/(NCT01469403) (28).

## Credit author statement

**J. Van den Langenbergh**: formal analysis, writing - original draft, writing - review & editing.

**Y.M. Bastiaansen-Jenniskens**: conceptualization, methodology, funding acquisition, project administration, data curation, validation, formal analysis.

**G.J.V.M. van Osch:** conceptualization, methodology, funding acquisition, project administration, writing - review & editing.

**J. Runhaar:** formal analysis, writing - review & editing.

**S.M.A. Bierma-Zeinstra**: formal analysis.

**K. Soballe:** conceptualization, methodology, funding acquisition, project administration.

**J. Laursen**: conceptualization, methodology, funding acquisition, project administration.

**A. Liljensoe**: conceptualization, methodology, funding acquisition, project administration, data curation, validation, formal analysis.

**N. Kops:** data curation, validation, formal analysis.

**I. Mechlenburg**: conceptualization, methodology, funding acquisition, project administration.

**S. Clockaerts**: supervision, conceptualization, methodology, funding acquisition, project administration, formal analysis, writing - original draft, formal analysis, writing - review & editing.

## Declaration of competing interest

The author(s) declared no potential conflicts of interest with respect to the research, authorship, and/or publication of this article.
