# Supplementary material for: PLOD2 gene expression in infrapatellar fat pad is correlated with fat mass in obese patients with end-stage knee osteoarthritis
Source: Osteoarthr Cartil Open. 2024 Apr 16;6(2):100469. doi: 10.1016/j.ocarto.2024.100469 (PMC11061337; doi:10.1016/j.ocarto.2024.100469)
Supplement: Multimedia component 1 [file mmc1.docx]

|  | **Age** |  | **Sex** |  | **BMI** |  | **Lean mass** | | **Fat mass** | | |  | **Glucose** | | **Total cholesterol** | | **LDL/HDL ratio** | | **TGLY** |  | **Leptin** |  |
| --- | --- | --- | --- | --- | --- | --- | --- | --- | --- | --- | --- | --- | --- | --- | --- | --- | --- | --- | --- | --- | --- | --- |
| **Age** | r = 1.00 | / | r = -0.27 | P = 0.06 | r = -0.28 | P = 0.32 | r = 0.33 | P = 0.30 | r = -0.34 | P = 0.31 | r = -0.11 | | | P = 0.27 | r = -0.20 | P = 0.85 | r = -0.08 | P = 0.89 | r = -0.09 | P = 0.76 | r = -0.26 | P = 0.36 |
| **Sex** | r = -0.27 | P = 0.06 | r = 1.00 | / | r = 0.43 | P = 0.43 | r = -0.89 | P = 0.26 | r = 0.89 | P = 0.24 | r = 0.15 | | | P = 0.71 | r = 0.11 | P = 0.23 | r = 0.01 | P = 0.61 | r = 0.19 | P = 0.35 | r = 0.53 | P = 0.36 |
| **BMI** | r = -0.28 | P = 0.32 | r = 0.43 | P = 0.43 | r = 1.00 | / | r = -0.48 | P = < 0.001 | r = 0.50 | P = < 0.001 | r = 0.17 | | | P = 0.25 | r = 0.17 | P = 0.25 | r = 0.21 | P = 0.18 | r = 0.41 | P = < 0.001 | r = 0.71 | P = < 0.001 |
| **Lean mass** | r = 0.33 | P = 0.30 | r = -0.89 | P = 0.26 | r = -0.48 | P = < 0.001 | r = 1.00 | / | r = -0.99 | P = < 0.001 | r = -0.20 | | | P = 0.18 | r = -0.31 | P = 0.03 | r = -0.12 | P = 0.42 | r = -0.35 | P = 0.02 | r = -0.61 | P = < 0.001 |
| **Fat mass** | r = -0.34 | P = 0.31 | r = 0.89 | P = 0.24 | r = 0.50 | P = < 0.001 | r = -0.99 | P = < 0.001 | r = 1.00 | / | r = 0.21 | | | P = 0.16 | r = 0.30 | P = 0.04 | r = 0.12 | P = 0.42 | r = 0.35 | P = 0,02 | r = 0.63 | P = < 0.001 |
| **Glucose** | r = -0.12 | P = 0.27 | r = 0.15 | P = 0.71 | r = 0.17 | P = 0.25 | r = -0.20 | P = 0.18 | r = 0.26 | P = 0.16 | r = 1.00 | | | / | r = 0.07 | P = 0.64 | r = 0.03 | P = 0,83 | r = 0.39 | P = 0.01 | r = 0.28 | P = 0.07 |
| **Total**  **cholesterol** | r = -0.20 | P = 0.85 | r = 0.11 | P = 0.23 | r = 0.17 | P = 0.25 | r = -0.31 | P = 0.03 | r = 0.30 | P = 0.04 | r = 0.07 | | | P = 0.64 | r = 1.00 | / | r = 0.68 | P = < 0.001 | r = 0.41 | P = < 0.001 | r = 0.18 | P = 0.22 |
| **LDL/HDL ratio** | r = -0.08 | P = 0.89 | r = 0.01 | P = 0.61 | r = 0.21 | P = 0.18 | r = -0.12 | P = 0.42 | r = 0.12 | P = 0.42 | r = 0.03 | | | P = 0.83 | r = 0.68 | P = < 0.001 | r = 1.00 | / | r = 0.33 | P = 0.02 | r = -0.04 | P = 0.78 |
| **TGLY** | r = -0.09 | P = 0.76 | r = 0.19 | P = 0.35 | r = 0.41 | P = < 0.001 | r = -0.35 | P = 0.02 | r = 0.35 | P = 0.02 | r = 0.39 | | | P = 0.01 | r = 0.41 | P = < 0.001 | r = 0.33 | P = 0.02 | r = 1.00 | / | r = 0.33 | P = 0.03 |
| **Leptin** | r = -0.26 | P = 0.36 | r = 0.53 | P = 0.36 | r = 0.71 | P = < 0.001 | r = -0.62 | P = < 0.001 | r = 0.63 | P = < 0.001 | r = 0.28 | | | P = 0.07 | r = 0.18 | P = 0.22 | r = -0.04 | P = 0.78 | r = 0.33 | P = 0.03 | r = 1.00 | / |

*S1. Collinnearity plot: Pearson correlation coefficient (R) and P value on patient characteristics., LDL low density lipoprotein, HDL high density lipoprotein, TGLY triglcerides.*

| Gene | Forward oligonucleotides | Reverse oligonucleotides |
| --- | --- | --- |
|  |  |  |
| *PLOD2* | CCCTCCGATCAGAGATGATT | AATGTTTCCGGAGTAGGGGAGTCTTTTT |
| *COL1A1* | CAGCCGCTTCACCTACAGC | TTTTGTATTCAATCACTGTCTTGCC |
| *ASMA* | CGTTGCCCCTGAAGAGCAT | CCGCCTGGATAGCCACATACA |
| *TNFA* | GCCGCATCGCCGTCTCCTAC | AGCGCTGAGTCGGTCACCCT |
| *IL6* | TCGAGCCCACCGGGAACGAA | GCAGGGAGGGCAGCAGGCAA |
| *IL1B* | CCCTAAACAGATGAAGTGCTCCTT | GTAGTCGGATGCCGCCAT |
| *GADPH* | ATGGGGAAGGTGAAGGTCG | TAAAAGCAGCCCTGGTGACC |

S2. Gene primers and their corresponding forward and reversed oligonucleotides.
